# *Mycoplasma bovis* Infections: Occurrence, Pathogenesis, Diagnosis and Control, Including Prevention and Therapy

**DOI:** 10.3390/pathogens9120994

**Published:** 2020-11-27

**Authors:** Katarzyna Dudek, Ewelina Szacawa

**Affiliations:** Department of Cattle and Sheep Diseases, National Veterinary Research Institute, 57 Partyzantów Avenue, 24100 Pulawy, Poland; ewelina.szacawa@piwet.pulawy.pl

*Mycoplasma bovis* (*M. bovis*) is an etiological agent of bronchopneumonia, mastitis, arthritis, otitis, keratoconjunctivitis, meningitis, endocarditis and other disorders in cattle. It is known to spread worldwide, including countries for a long time considered free of the infection. This editorial summarizes the data described in the Special Issue entitled “*Mycoplasma bovis* Infections: Occurrence, Pathogenesis, Diagnosis and Control, Including Prevention and Therapy” consisting of eight research articles and a review. The research articles discuss the most important issues related to *Mycoplasma bovis* infections, including the lung local immunity in *M. bovis* pneumonia, antimicrobial susceptibility and antimicrobial resistance-associated genes of *M. bovis* isolates, *M. bovis* antibody testing, efficacy of seminal extender on *M. bovis* as well as imported bull examination for *M. bovis*, whereas the latest data were summarized in the review. 

The review of this Issue summarized the latest data on *Mycoplasma bovis* infections, introducing the problem, taking into account the issues related to spread of *M. bovis* around the world, the disease therapy and immunoprophylaxis of the infections. It discussed the current epizootic situation of *M. bovis*, including the studies from the countries for a long time considered free of *M. bovis*, such as Finland, New Zealand or Australia. The review listed the most important courses of *M. bovis* infection and their sources including colostrum, milk, air-borne, intrauterine and newly noticed semen. An important part of the review was also devoted to the description of currently used methods in the diagnosis of *M. bovis*, especially in terms of the specimen used. The review also addressed the issue of methods of the disease eradication and collected the most important recommendations in order to unify the rules of preventing *M. bovis* infections in the designed control programs [1]. 

The research article by Dudek et al. [2] described the leukocyte response in *M. bovis* pneumonia using the calf infection model. In the experimentally infected calves, the lung immune response manifested in both the T- and B-lymphocyte stimulation. The local immunity was also characterized by the increased phagocyte expression and upregulation of antigen-presenting mechanisms dependent on the MHC class II. On the other hand, the activation of peripheral antimicrobial mechanisms was manifested in the general stimulation of phagocytic activity and oxygen metabolism of leukocytes, however it depended on the stage of the disease. 

The work of Petersen et al. [3] aimed to compare two commercially available ELISAs for *M. bovis* antibody detection in adult cows from 12 dairy herds with a known previous *M. bovis* infection status. With the use of the newly commercially released ELISA, more positive serum and milk samples were diagnosed compared to the second of the tested tests, which proved its higher sensitivity. Additional analysis of the concordance correlation coefficient of sample-to-positive percentage showed high comparability between the serum and milk samples for this test; however, with the higher serum values. These results indicate that the milk samples are a good matrix for *M. bovis* antibody testing in this test as the serum samples and can be used as a replacer. As a result of this study, the suitability of the newly commercially released ELISA for the evaluation of subclinically infected animals and bull tank milk samples as well as for herd-level control was proposed. However, the specificity of this test was questioned, which may be related to cross-reactions presence. In the authors’ opinion, the second of the tested tests seems to be useful primarily for detection of clinically ill animals.

The research article by Catania et al. [4] discussed the role of newly imported bulls in spreading of bovine mycoplasmas in fattening farms, including *M. bovis*. In 19.1% of total of 711 nasal swabs three times collected (on arrival, at 15 and 60 days after arrival), *M. bovis* was isolated as poor or mixed cultures with other species of the *Mollicutes* class. The results showed a clear dependence of *M. bovis* prevalence on the sampling time. On arrival, the majority of bulls tested were free of *M. bovis*. Significantly increased *M. bovis* prevalence was observed 15 days after arrival which ranged between 40 and 81% dependent on the method used, whereas general its decrease was noted 45 days after. Here, there was also no predictive role of environmental conditions in *M. bovis* prevalence in the imported bulls. 

The study of Pohjanvirta et al. [5] drew attention to the real risk of *M. bovis* transmission via artificial insemination in the context of the poor mycoplasmacidal efficacy of antibiotics used in the semen extender. The efficacy of the combinations of antibiotics added to the semen extender used in this study was dependent on the *M. bovis* concentration in spiked semen samples and differed in the case of the two tested bacterial strains, ATCC and wild type. Additionally, from all three tested DNA extraction methods, the one with the highest sensitivity for detection of either of the *M. bovis* strains in the pools spiked with low concentration of the pathogen was selected. To prevent the transmission of *M. bovis* via the contaminated semen, the authors suggested using a higher than recommended combination of antibiotics added to the semen extender, or which would be the best solution to test bulls intended for artificial insemination for *M. bovis* and use semen free of the pathogen.

Ledger et al. [6] covered the topic in the field of increasing resistance of *M. bovis* isolates for antimicrobials that was reported in many countries. This article describes the antimicrobial resistance-associated genes in *M. bovis* isolate from 2019 that had high minimum inhibitory concentration (MIC) for fluorochinolones, tetracyclines, macrolides, lincosamides and pleuromutulins. With the use of whole genome sequencing (WGS) more non-synonymous mutations and gene disruptions were identified in the recently received *M. bovis* isolate when compared with the past isolate and reference strain PG45. The researchers selected 55 genes for the potential function of antimicrobial resistance. It gives the possibility to further analyze this candidate AMR genes and compare it with another research in the future. 

The main aim of the work of Kinnear et al. [7] was to assess the relationship between the genotypes and phenotypes of *M. bovis* isolates in the evaluation of antimicrobial resistance to macrolides, used both in the prevention and treatment of *M. bovis* infections in feedlot cattle. In this cross-sectional twelve-year study a total of 126 *M. bovis* isolates were tested. The samples originated from feedlot cattle of different health status and were collected from multiple anatomical locations. The MIC values for five selected macrolides were estimated following the antimicrobial susceptibility testing. Additionally, the genotype of all isolates based on the number and positions of single nucleotide polymorphisms (mutations) in the 23S rRNA gene alleles and ribosomal proteins was determined. The efficacy of the examined macrolides was depended on the type of mutations determined for each *M. bovis* isolate, with exception of tildipirosin and tilmicosin, which, according to the authors, seem to be unsuitable for *M. bovis* infection treatment in cattle.

The two-year study of Becker et al. [8] concerned longitudinal monitoring of *M. bovis* infections in 25 feedlots. It revealed that the low *M. bovis* prevalence was observed in calves at their arrival in the feedlot, whereas the high prevalence was seen 4 weeks after the antimicrobial treatment. This at indicates the ineffective antimicrobial treatment of the infected calves due to antibiotic resistance of *M. bovis* strains. The important finding was that these strains were resistant to antibiotics prior to any treatments of the calves and it led to the clinical recovery of animals without *M. bovis* clearance. This research supports the previous finding about the overall multiresistance of *M. bovis* isolates to the most of the tested antimicrobials except for fluoroquinolones and that the most strains belonged to little variable subtype ST2, based on the single-locus sequence analysis of *polC* gene.

García-Galán et al. [9] described the research on *M. bovis* isolated from beef and dairy cattle. According to the study, this pathogen was present in 40.9% of examined beef cattle and in 16.36% of dairy cattle. The MIC testing and WGS results showed that the most isolates were resistant to many antimicrobials (macrolides, lincosamides and tetracyclines). The genome sequencing also revealed that the *M. bovis* isolates belonged to only two STs (ST2 and ST3). The research revealed that the most isolates that belonged to ST3 had high MIC values for fluoroquinolones and the ST2 isolates had lower MIC values for this group of antimicrobials. The researchers also showed that the main differences between the ST2 and ST3 were located in the quinolone-resistance determining regions of *GyrA* and *ParC* genes. The mutations in these genes were found only in the *M. bovis* isolates belonged to ST3. In vitro testing revealed that only valnemulin was effective against the *M. bovis* isolates from both STs.

The articles included in this Special Issue present the most up-to-date data on *M. bovis* infections, including the disease pathogenesis and therapy, and contribute significantly to improving knowledge in this field.
